# Emerging functions of piwi‐interacting RNAs in diseases

**DOI:** 10.1111/jcmm.16466

**Published:** 2021-05-04

**Authors:** Kai Wang, Tao Wang, Xiang‐Qian Gao, Xin‐Zhe Chen, Fei Wang, Lu‐Yu Zhou

**Affiliations:** ^1^ Institute for Translational Medicine The Affiliated Hospital of Qingdao University College of Medicine Qingdao University Qingdao China

**Keywords:** ageing, biomarker, cancer, neurodegenerative diseases, PIWI‐interacting RNAs

## Abstract

PIWI‐interacting RNAs (piRNAs) are recently discovered small non‐coding RNAs consisting of 24‐35 nucleotides, usually including a characteristic 5‐terminal uridine and an adenosine at position 10. PIWI proteins can specifically bind to the unique structure of the 3′ end of piRNAs. In the past, it was thought that piRNAs existed only in the reproductive system, but recently, it was reported that piRNAs are also expressed in several other human tissues with tissue specificity. Growing evidence shows that piRNAs and PIWI proteins are abnormally expressed in various diseases, including cancers, neurodegenerative diseases and ageing, and may be potential biomarkers and therapeutic targets. This review aims to discuss the current research status regarding piRNA biogenetic processes, functions, mechanisms and emerging roles in various diseases.

## 1 INTRODUCTION

At present, countries around the world are facing the trend of an ageing population. With the increase in harmful substances in the environment, modern humans have to face an increasing number of diseases. It was reported that there were 18.1 million new cancer cases (9.5 million males and 8.6 million females) and 9.6 million deaths (5.4 million males and 4.2 million females) worldwide in 2018, further increasing the global cancer burden. Due to relatively late disease detection and high metastasis and recurrence rates, treatment is often ineffective,^1^ highlighting the necessity of new biomarkers for cancer diagnosis and prognosis and new targets for effective treatments. Ageing and cardiovascular disease also plague human health. Cardiovascular disease is increasingly endangering human life and health. Basic life sciences research on cardiovascular disease is of great and far‐reaching significance for the survival and development of human society. Many studies have found that piRNAs in the heart have numerous potential regulatory effects that need to be further explored.^2^

RNA interference (RNAi) was discovered in the late 1990s,^3^ and it significantly changed our understanding of the regulation of gene expression. These non‐coding RNAs (ncRNAs) are not translated into proteins but instead work through pairing with complementary bases of targeted RNA or binding to targeted proteins.^4^ In 2006, Aravin et al^5^ isolated a small RNA from the vas deferens of 3‐month‐old C57 mice and found that the highly expressed small RNA interacted with MILI (a PIWI subprotein); they named this small RNA piRNA. During the study of piRNA biosynthesis, it was found that a large number of piRNA clusters in the intergenic region were transcribed to form piRNA precursors by bidirectional or unidirectional transcription. Endonucleases then digested these piRNA precursors to produce piRNAs. Additionally, piRNAs can also be produced in the mRNA 3′ untranslated region (3′ UTR) and some long non‐coding regions in the genome.^6^ Sixteen years have passed since the first discovery of piRNAs, but our understanding of the functions of piRNAs and PIWI proteins remains unclear. In this review, we discussed the current research status regarding piRNA biogenetic processes, functions and mechanisms, along with their roles in diseases.

## 2 BIOGENESIS OF PIRNAS

**FIGURE 1:**
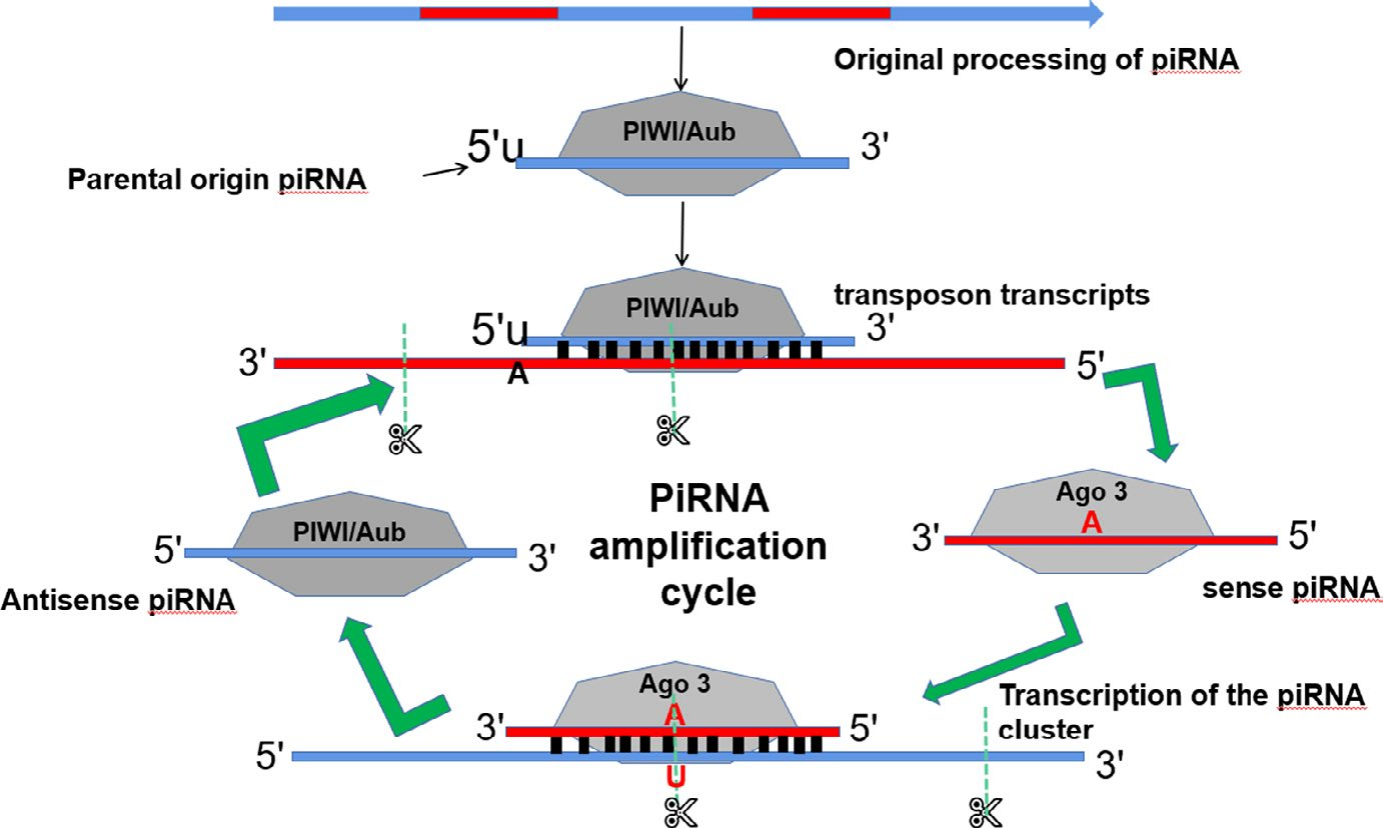
The mechanism of piRNA production and amplification. The piRNAs that are related to PIWI and Aub are usually antisense and complementary to transposon transcripts. PIWI/Aub starts from the 5′ end of the antisense piRNA and cuts transposon transcripts between 10 and 11 nt to generate Ago3‐related piRNA. Ago3 identifies piRNA cluster transcripts and generates more PIWI/Aub‐related antisense strand piRNAs

Newly transcribed piRNAs are usually transported out of the nucleus, processed and matured in the cytoplasm, and then perform their biological function. The mechanism of piRNA production is divided into the primary and secondary piRNA pathways, which mainly revolves around the ping‐pong amplification cycle process (Figure 1).^7^ In a further study of the piRNA ping‐pong mechanism, it was found that the piRNA synthesis pathway exists not only in animal germ cells but also in somatic cells and that there are some differences in the piRNA ping‐pong mechanism process between germ cells and somatic cells.^8^, ^9^

**FIGURE 2:**
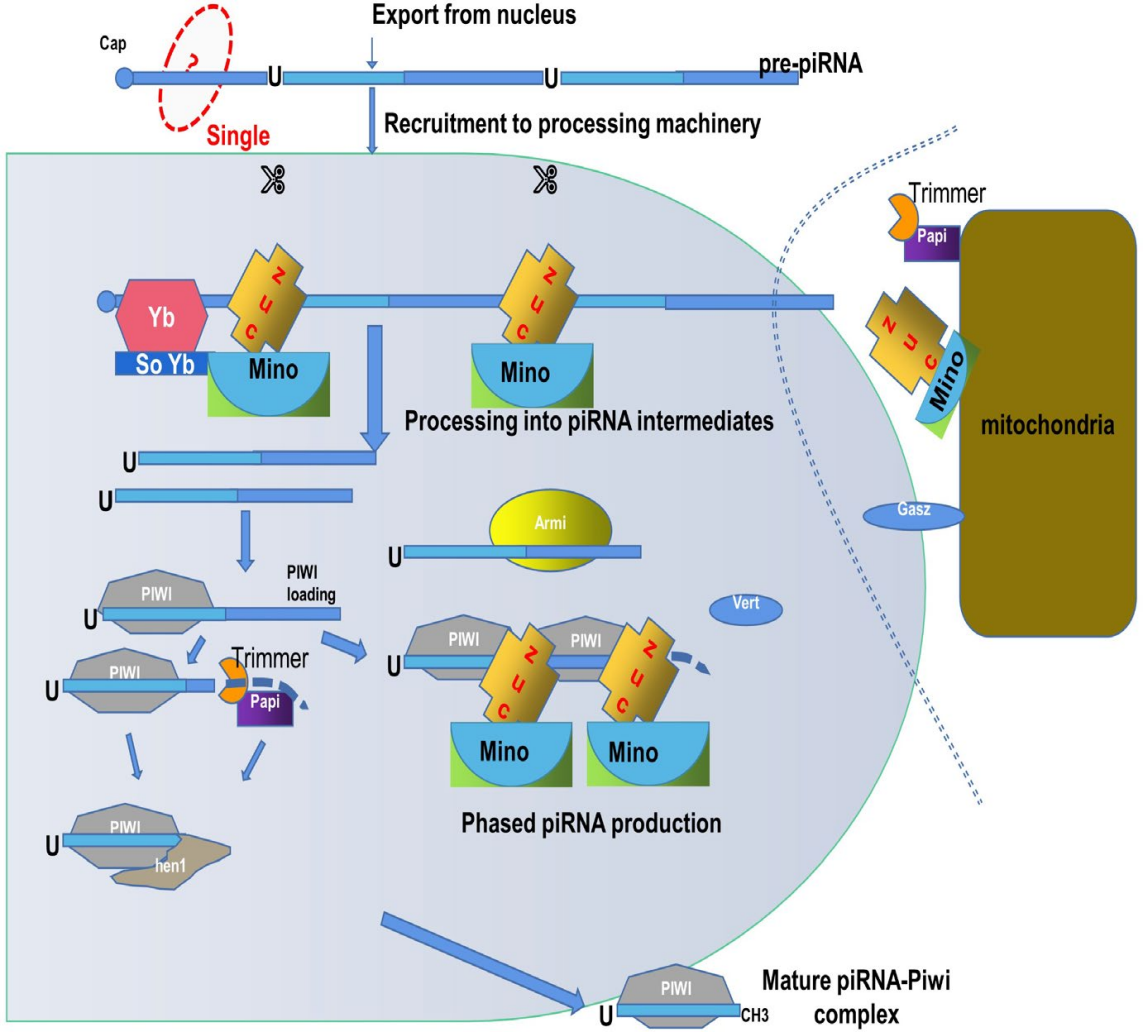
Hen1‐mediated piRNA generation pathway. After transporting out of the nucleus, the piRNA precursor (with the red ‘?’) is transferred and processed by Yb. Then, Zuc and its cofactors interact to produce piRNA intermediates. This processing step also requires Vret, Mino and Gasz. Armi seems to be involved in the decomposition of the secondary structure. After that, piRNA intermediates are loaded into PIWI. Zuc cleaves the piRNA and forms the 3′ end. Trimmer and its cofactor Papi participate in removing the piRNA intermediate and lead to the formation of the piRNA‐PIWI complex. The piRNA‐PIWI complex becomes mature after Hen1 methylation. Factors that participate in the biogenesis of newborn piRNAs are located on the outer membrane of mitochondria

The piRNA somatic pathway can directly rely on PIWI proteins and piRNA clusters of flamenco fragments to produce piRNAs. First, the piRNA precursor binds to the cytoplasmic Yb body to form the 5′ U site of the intermediate piRNA under the action of Zucchini (Zuc) and cofactors (Vret, Mino, Gasz, etc).^10^ The secondary structure of the piRNA is generated, and Zuc cleaves the PIWI‐piRNA sequence to form a 3′ end. The exonuclease trimer and Papi cleave and further mature the PIWI‐piRNA.^11^ Then, Hen1‐mediated methylation occurs, and finally, the mature PIWI‐piRNA complex enters the nucleus to play a silencing role. Extracellular factors of mitochondria also play an important role in this pathway (Figure 2).^8^

## 3 CHARACTERISTICS OF PIRNAS

Compared with siRNAs and miRNAs, piRNAs are greater in number, with approximately 5 × 10^4^ species. The length is slightly longer than the first two, generally within the range of 24~35 nt. They are methylated at the 3′ end, the first nucleotide at the 5′ end is typically uridine, and the 10th base is typically adenine. In mice, piRNAs are mainly distributed on the X chromosome and rarely on the Y chromosome, but most piRNAs in the human body are distributed on autosomal chromosomes and are rarely on sex chromosomes.^12^ In Table 1, we summarize the many kinds of small non‐coding RNAs according to their differences in size and function.

**TABLE 1:** Differences between various non‐coding small RNAs

Types	Size	Function	References
miRNAs	20‐23(nt)	Binds to the 3′ untranslated region of the target mRNA; causes the degradation or translation inhibition of the target mRNA; participates in the regulation of post‐transcriptional gene expression in animals and plants	[36]
siRNAs	20‐25(nt)	Processed by Dicer enzyme; the main member of siRISC; stimulates the silencing of the complementary target mRNA	[37]
snRNAs	100‐215(nt)	The main component of RNA spliceosome in eukaryotic post‐transcriptional processing and participates in the processing of mRNA precursors	[38]
snoRNAs	60‐130(nt)	Plays a role in the biosynthesis of ribosomal RNA and guides the post‐transcriptional modification of snRNA, tRNA and mRNA	[39]
tsRNAs	18‐40(nt)	A classical regulatory small non‐coding RNA involved in a variety of physiological and pathological processes	[40]
piRNAs	24‐35(nt)	Mainly exists in mammalian germ cells and stem cells; regulates the gene silencing pathway by combining Piwi subfamily proteins to form the piRNA complex (piRC)	[41]

## 4 MECHANISM AND FUNCTION OF PIRNAS

### 4.1 piRNAs participate in transcriptional silencing

**FIGURE 3:**
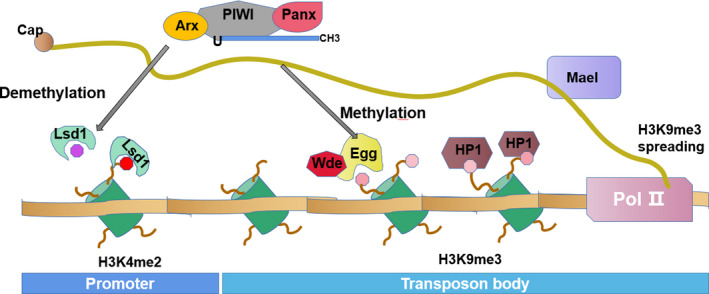
piRNA participates in methylation regulation. The complex that includes piRNA, PIWI, Arx and Panx induces cotranscriptional inhibition. Then, the targeted transposon will be labelled with histone 3 lysine 9 trimethylation (H3K9me3), which is a modification produced by anovulatory (Egg) and its cofactor Windei (Wde). The subsequent recruitment of H3K9me3 by HP1 leads to the formation of heterochromatin. Lysine‐specific demethylase 1 (Lsd1) may remove active histone 3 lysine 4 dimethylation (H3K4me2) labelling in the transposon promoter region, thus effectively inhibiting transposons at the transcriptional level. Maelstrom (Mael) blocks the transmission of H3K9me3

Previous studies on small RNA‐mediated transcriptional gene silencing (TGS) in plants and yeasts have shown that the existence of nuclear PIWI proteins, and various coincidences of inhibitory histone labelling or DNA methylation in some piRNA pathway mutants, eventually lead to PIWI‐dependent transcriptional silencing in animals.^13^ However, as research continues, the direct pathways of transposon silencing in the piRNA pathway have also been found.^14^, ^15^ Among them, PIWI proteins mainly act at the transcriptional level (Figure 3). Studies suggest that mature piRNA‐PIWI/Asterix (Arx) complexes scan for and detect nascent transposon transcription.^8^, ^16^ After detection, transcriptional inhibition is enforced by the formation of heterochromatin. In addition, it is speculated that piRNAs inhibit the insertion of foreign sequence fragments.^17^

### 4.2 piRNAs participate in post‐transcriptional gene silencing (PTGS)

Many piRNAs regulate the post‐transcriptional network to inhibit target function through piRNA‐RNA interactions, and the mechanism is similar to that of miRNAs.^18^, ^19^ These RNA packets include transcribed pseudogenes,^20^ long non‐coding RNAs (lncRNAs),^21^ and mRNAs.^22^ The piRNA interaction requires base pairing at the 5′ end of the piRNA, which exhibits strict base pairing in the 2‐11 nt range and loose base pairing in the 12‐21 nt range.^23^ The piRNA‐PIWI complex recruits carbon catabolite‐repressed 4‐negative TATA‐less (CCR4‐NOT) and Smaug (SMG) to form specific pi‐RISCs, which can promote RNA inhibition through imperfect base pairing through miRNA‐like mechanisms.^24^, ^25^, ^26^

The piRNA‐PIWI ribonucleoprotein complex can also lead to transposable element post‐transcriptional silencing, thus maintaining the integrity of the genome.^25^ This transposable element post‐transcriptional silencing can drive genome evolution and must be tightly regulated, as overactivity is detrimental to the host.^27^ In ping‐pong piRNA amplification, the mature nucleoprotein complex modified by symmetrical dimethylarginine (SDMA) is recruited by Krimper, and it also interacts with unloaded Ago3, thus binding these together.^8^ Since both the complex and Ago3 have PIWI domains with RNase H endonuclease activity, the newly established complex can selectively detect and cleave transposon RNA so that transposable elements (TEs) are silenced at the post‐transcriptional level.^8^

### 4.3 piRNA regulates protein and gene expression

piRNAs are regulators of proteins and genes.^19^ The piRNA/PIWI complex binds directly to some proteins through piRNAs or the PIWI protein PAZ domain.^18^ For example, piRNA‐54265 can bind to the PIWIL2 protein and promote the formation of the PIWIL2/STAT3/phosphorylated SRC (p‐SRC) complex, activating STAT3 signalling and promoting the proliferation, metastasis and chemotherapy resistance of colorectal cancer cells.^28^ This interaction promotes the interaction of multiple proteins and changes their subcellular localization. SEPW1P is the reverse transcriptional pseudogene of SEPW1. piRNA‐36712 can compete with RNA produced by SEPW1P (SEPW1P RNA) for miR‐7 and miR‐324 and finally inhibit the expression of SEPW1 mRNA.^20^

## 5 RESEARCH ON PIRNAS IN DISEASES

### 5.1 piRNAs in cancer

Although recent studies have found that piRNA expression in somatic cells is relatively low, many piRNAs are involved in tumour occurrence and development (cancer cell proliferation, apoptosis, metastasis and invasion). These piRNAs are dysregulated in tumour tissue and play roles in tumour promotion or tumour inhibition. To stimulate more research to fully understand the molecular biological mechanisms of piRNAs in tumour diseases, we summarize some recent studies on piRNAs in multiple cancers for reference (Table 2).

**TABLE 2:** The role of piRNAs in various cancer

Tumour type	piRNA	Interaction	References
Alcoholic nasopharyngeal carcinoma	piR‐35373; piR‐266308; piR‐58510; piR‐38034	Expression disordered after drinking	[42]
Bladder cancer	piRABC (DQ594040)	Affected the expression of TNFSF4 protein and played an important role in the development of bladder cancer	[43]
Breast cancer	piR‐21285	Functioned in the occurrence and development of breast cancer through related epigenetic mechanisms	[44]
	piR‐4987; piR‐20365; piR‐20485; piR‐20582	Up‐regulated in breast cancer and may be used as a biomarker of breast cancer	[45]
	piRNA‐36712	A new tumour suppressor gene that can be used as a promising predictor of breast cancer prognosis	[46]
	piR‐36026	Plays a role in the regulation of tumour suppressor genes and mediates the progression of breast cancer in vivo and in vitro	[47]
	piR‐016658; piR‐016975	Associated with tumour initiation and progression	[48]
Colorectal cancer	piR‐15551	Produced by LNC00964‐3 and participates in the occurrence and development of colorectal cancer	[49]
	piR‐1245	Targeted tumour suppressor gene has a carcinogenic effect and can be used as a potential prognostic biomarker of colorectal cancer	[50]
	piR‐5937; piR‐28876	Can be used as a potential biomarker for early detection of colon cancer	[51]
	piR‐54265	By promoting the formation of PIWIL2/STAT3/phosphorylated SRC (p‐SRC) complex, activates the STAT3 signal pathway, promoting the proliferation, metastasis and chemotherapy resistance of colorectal cancer cells, thus playing a carcinogenic role; may become a therapeutic target for colorectal cancer	[28]
	piR‐020619; piR‐020450	Has potential as a specific marker for early detection of colorectal cancer	[10]
	piR‐24000	High expression of PIR‐24000 was significantly associated with aggressive colorectal cancer phenotypes	[52]
Oesophageal cancer (EC)	piR‐823	The level of piR‐823 was significantly associated with lymph node metastasis	[10]
Fibrosarcoma	piR‐39980	Has a strong antitumor effect	[53]
Gastric cancer (GC)	piR‐651; piR‐823	Can be used as a valuable biomarker for detecting circulating gastric cancer cells	[54, 55
	piR‐59056; piR‐32105; piR‐58099	Could be used as tumour markers in gastric cancer, furthermore, could effectively stratify GC patients into low‐ and high‐risk recurrence groups	[56]
	piR‐1245	Associated with overall survival (OS) and progression‐free survival (PFS)	[57]
Glioblastoma	piR‐8041	Inhibits cell proliferation, induce cell cycle arrest and apoptosis, and inhibit cell survival pathway	[58]
Hepatocellular carcinoma	piR‐Hep1	Silencing piR‐Hep1 inhibits the viability and invasiveness of cells and may lead to a decrease in the level of phosphorylation of active AKT	[59]
Lung cancer (LC)	piR‐34871; piR‐52200	Correlated with RASSF1C expression; promoted cell proliferation and colony formation by reducing AMPK phosphorylation of the ATM‐AMPK‐p53‐p21cip pathway	[60]
	piR‐55490	Inhibits the activation of Akt/mTOR pathway and inhibits the growth of lung cancer	[22]
	piR‐651	Inhibits cell proliferation, migration and invasion and induces apoptosis, thereby regulating the carcinogenic activity of NSCLC	[54]
	piRNA/piRNA‐L	Interaction with proteins under pathophysiological conditions	[61]
Multiple myeloma (MM)	piRNA‐823	Promoted angiogenesis and played a carcinogenic role in MM	[62]
	piR‐004800	Participates in the carcinogenesis of the PI3K/AKT/mTOR pathway	[63]
Ovarian cancer	piR‐33733	Inhibits apoptosis by binding to targeted mRNA	[64]
	piR‐52207	Promotes cell proliferation, migration and tumorigenesis by binding to targeted mRNA	[64]
Osteosarcoma (OS)	piR‐39980	Related to the ability of cell migration and invasion	[65]
Oral squamous cell carcinoma (OSCC)	piR‐1037	Enhances the chemoresistance and motility of OSCC cells	[66]
Pancreatic cancer	piR‐017061	Expression is down‐regulated in cancer cells	[67]
Prostate cancer	piR‐31470	Increased vulnerability to oxidative stress and DNA damage in human prostate epithelial RWPE1 cells.	[68]
Thyroid cancer (TC)	piR‐13643; piR‐21238	Expected to be a new biomarker for accurate detection of PTC.	[69]
Urinary tumours	piR‐32051; piR‐39894; piR43607	Highly associated with clear cell renal cell carcinoma (ccRCC) metastasis, late clinical‐stage and poor cancer‐specific survival	[67]
	piR‐823	Detection of piR‐823 in urine is helpful for the diagnosis of renal cell carcinoma	[62]
	piR‐57125; piR‐30924; piR‐38756	Abnormal expression in renal cell carcinoma can be used as a potential biomarker to judge the prognosis of renal cell carcinoma	[70]

### 5.2 piRNAs in ageing

piRNAs can maintain genome integrity through the PIWI‐piRNA pathway, which plays an important role in ageing. TEs, also known as ‘jumping genes’, can move from one genome site to another, resulting in insertion mutations.^29^ With organism ageing, TEs become increasingly active and multiply in the somatic cell genome. These TE characteristics highlight their decisive mutagenic role in the gradual decomposition of genetic information, a molecular marker related to ageing.^30^ Therefore, TE‐mediated genomic instability may greatly promote the ageing process. The PIWI‐piRNA pathway can inhibit the activity of TEs and then delay ageing to a certain extent.^6^ Studies by Sousa‐Victor et al^31^ have shown that knockout of the PIWI gene in Drosophila intestinal stem cells (ISCs) can damage intestinal regeneration and lead to the loss of ISCs and their offspring due to apoptosis. Studies have also shown that PIWI expression is sufficient to reduce age‐related retrotransposon expression, DNA damage, phenotypic misdifferentiation and apoptosis.^31^

### 5.3 piRNAs in the heart

An increasing number of studies have found that piRNAs exist in many somatic cells in addition to germ cells, including the heart, which may be related to many heart‐related pathophysiological processes.^2^ piRNAs are expressed during the process of cardiomyocyte differentiation. Their expression levels change during different developmental stages.^32^ Additionally, piRNAs exist in cardiac progenitor cells,^33^ suggesting that piRNA may play an important role in the process of heart regeneration and participate in the maintenance and differentiation of cardiomyocytes. It has also been found that piRNAs exist in the hypertrophied heart.^34^ There is also a significant expression of piRNA in the serum exosomes of patients with heart failure. piRNAs might also be potential factors in markers of heart failure.^35^

## 6 PIRNA ONLINE DATABASES

Researchers may need to refer to many resources to better design their experiments and choose appropriate research models before carrying out piRNA projects. To facilitate future research, we collated free online databases to provide valuable piRNA information (Table 3). There is not yet a single fully featured database, so researchers should make use of each database according to their different functional features.

**TABLE 3:** piRNA online databases

Database	URL	Function	References
piRDisease v1.0	http://www.piwirna2disease.org/index.php	Provides a large number of experimentally proven piRNAs related to various diseases	[71]
piRNAQuest	http://bicresources.jcbose.ac.in/zhumur/pirnaquest/	Provides piRNA annotations based on their localization in gene, intron, intergenic, CDS, 5′ UTR, 3′ UTR and repetitive regions	[72]
piRBase V2.0	http://www.regulatoryrna.org/database/piRNA/	Systematically integrates epigenetic and post‐transcriptional regulation data to support piRNA functional analysis	[73, 74
IsopiRBank	http://mcg.ustc.edu.cn/bsc/isopir/index.html	Users can select isoforms of interest for further analysis, including target prediction and enrichment analysis	[75]
PVsiRNAdb	http://www.nipgr.res.in/PVsiRNAdb	Provides a resource for transcriptional regulatory information of RNA interference	[76]
piRNA cluster database	http://www.smallrnagroup‐mainz.de/piRNAclusterDB.html	Provides comprehensive data on piRNA clusters in multiple species, tissues and developmental stages	[77]
piRTarBase	http://cosbi6.ee.ncku.edu.tw/piRTarBase/	Predicts binding sites of piRNAs to miRNAs	[78]

## 7 DISCUSSION

piRNAs are recently discovered small non‐coding RNAs with flexible functions. With the development of bioinformatics and high‐throughput sequencing technology, the gene regulation function of piRNAs has become increasingly important. Current studies have found an abnormal expression of piRNA in the progression of diseases, but the specific molecular mechanism of piRNA requires further studies. There is a little research or application of piRNAs in targeted therapy. Therefore, we hope to build a relatively new knowledge network to explain the biogenesis and function of piRNAs and their relationships with related diseases, hoping to identify common targets among age‐related diseases and shed new light on their clinical application.

## CONFLICT OF INTEREST

The authors have no conflicts of interest to declare.

## AUTHOR CONTRIBUTION

**Kai Wang:** Data curation (equal); Formal analysis (equal); Methodology (equal); Writing‐original draft (lead). **Tao Wang:** Investigation (equal). **Xiangqian Gao:** Formal analysis (equal); Visualization (supporting). **Xinzhe Chen:** Investigation (equal); Writing‐original draft (equal). **Fei Wang:** Formal analysis (equal); Writing‐original draft (equal). **Luyu Zhou:** Data curation (lead); Formal analysis (lead); Funding acquisition (lead); Investigation (lead); Project administration (lead); Resources (lead); Supervision (lead); Validation (lead); Writing‐review & editing (lead).

## Data Availability

Data sharing is not applicable to this article as no new data were created or analysed in this study.
